# Clinical Responses to Rituximab in a Case of Neuroblastoma with Refractory Opsoclonus Myoclonus Ataxia Syndrome

**DOI:** 10.1155/2012/164082

**Published:** 2012-11-06

**Authors:** Samin Alavi, Ali Kord Valeshabad, Borhan Moradveisi, Ali Aminasnafi, Mohammad Taghi Arzanian

**Affiliations:** ^1^Pediatric Hematology and Oncology Department, Mofid Children's Hospital, Shahid Beheshti University of Medical Sciences, Tehran 15468-15514, Iran; ^2^Division of Gastroenterology and Hepatology, Johns Hopkins University, Baltimore, MD 21218, USA

## Abstract

Opsoclonus myoclonus ataxia syndrome (OMS) is a rare neurologic syndrome. In a high proportion of children, it is associated with neuroblastoma. The etiology of this condition is thought to be immune mediated. In children, immunotherapy with conventional treatments such as corticosteroids, intravenous immunoglobulin, adrenocorticotropic hormone, and even antiepileptic drugs has been tried. Recently rituximab has been used safely for refractory OMS in children with neuroblastoma. Our patient was a 3.5-year-old girl referred for ataxia and dancing eye movements starting since 1.5 years ago. She was diagnosed with neuroblastoma on imaging studies on admission. The OMS was refractory to surgical resection, chemotherapy, corticosteroids, and intravenous immunoglobulin. Patient received rituximab simultaneously with chemotherapy. The total severity score decreased by 61.1% after rituximab. Patient's ataxia markedly improved that she was able to walk independently after 6 months. Our case confirmed the clinical efficacy and safety of rituximab in a refractory case of OMS.

## 1. Introduction

Opsoclonus myoclonus ataxia syndrome (OMS) is a serious, but rare immune-mediated paraneoplastic syndrome with an incidence rate of 0.18 cases per million population per year which occurs in 1.8% to 3% of patients with neuroblastoma [1, 2]. Despite the favorable prognosis of patients with OMS associated with neuroblastoma after cure of their cancer, considerable proportions of patients are refractory and suffer long-term sequelae of this syndrome [3–6]. The recent concern is now focused to find an appropriate treatment modality for those refractory cases who do not respond to conventional treatments.

A considerable number of recent investigations confirmed OMS as a humorally mediated disease which may respond to immune suppression therapies especially those targeted to B-cell lymphocytes [7]. Rituximab, a monoclonal anti-CD20 antibody, has been reported to have favorable effects to subset refractory signs of OMS [8–12]. However, rituximab was approved for treatment of B cell non-Hodgkin lymphoma [13], it has been used for other types of autoimmune disorders [14–16]. Here we investigate the clinical responses to rituximab in a case of refractory OMS who did not respond to surgical resection of tumor and standard chemotherapy.

## 2. Case

A 3.5-year-old girl was referred to pediatric neurology department with ataxia and dancing eye movements starting since 1.5 years ago. The patient was admitted for further evaluations. Initial laboratory workups including hematological and biochemical parameters were in normal range. The 24-hour urinary vanillin mandelic acid (VMA) concentration showed an increase. Imaging studies including chest and abdominal computed tomography (CT) scan revealed a tumoral lesion located in posterior mediastinum extending from T6 to T10. The patient was scheduled for thoracotomy and the mass was resected as much as possible.

Surgical specimens were submitted to the histopathologic laboratory. Microscopic evaluation was compatible with poor stroma, undifferentiated neuroblastoma. N-myc was amplified to more than 10 copy numbers using fluorescence in situ hybridization (FISH). Bone survey study was performed and show no abnormality. Bone marrow aspiration and whole body Tc-99 m was unremarkable and brain CT scan was normal.

Chemotherapy started referring to N6 protocol [17]. Within the first weeks following chemotherapy patient's symptoms and signs remarkably alleviated except opsoclonus-myoclonus syndrome which was the main complaint of the patient. It was planned to prescribe corticosteroids and clonazepam to treat the syndrome. After about 4 weeks, the debilitating problem for the patient was ataxia which showed no acceptable improvement. It was decided to prescribe intravenous immunoglobulin (IVIG) 1 g/Kg, monthly to improve ataxia and opsoclonus. After 2 courses of IVIG for 2 consecutive months, no obvious change was observed. Finally rituximab 375 mg/m^2^ as the last recognized option was considered to control patient's symptoms.

OMS Evaluation Scale was applied to evaluate clinical outcome of the patient as previously described [18]. A significant improvement in patient's ataxia and eye movements was observed after first dose of rituximab. About 3-4 weeks later the symptoms returned but with much less severity. Thus, it was decided to continue rituximab at least for 4 doses while patient was under close observation for clinical improvement. As it is shown in Table 1, based on the OMS Evaluation Scale, total severity score decreased by 61.1% after rituximab (28/36 versus 6/36). After each administration of rituximab the patient showed marked improvement; however, the symptoms again reoccurred exactly close to the next chemotherapy cycle, each time milder than previous. Patient's ataxia markedly improved to the extent that she was able to walk almost independently after 6 months. Meanwhile, it is noteworthy that the patient was in remission for her tumor at this time. The follow-up imaging studies were normal and there was no tumoral lesion after 1 year of treatment. N-Myc amplification decreased to the normal range. Considering the patient as stage 3 she was scheduled for autologous stem cell transplantation.

## 3. Discussion

For our patient, 4 weekly doses of rituximab were associated with marked clinical improvement in a refractory case to conventional treatments and also chemotherapy. Although corticosteroids and adrenocorticotropic hormone (ACTH) have been described as “gold standard” treatment for OMS [6], approximately 80% of treated patients develop long-term neurological sequelae [3]. Moreover, the relapse rate with conventional treatment is reported 75% [6, 19]; this rate will increase when facing refractory cases. Long-term prescription of corticosteroids and/or ACTH would be also associated with drug side effects which restrict continuation of these treatments. Thus, there has been a trend to explore further treatment options for OMS patients, especially in refractory cases.

Various combinations of drugs have been reported for treatment of OMS. Favorable outcomes have been reported for combination of dexamethasone and cyclophosphamide [6], cyclophosphamide and chemotherapy [2, 4], combination of ACTH, IVIG and rituximab [18], and also monotherapy with rituximab [8–11]. Ertle et al. demonstrated high-dose dexamethasone pulses as an effective therapy for OMS even in cases in whom previous corticosteroid, rituximab, and cytostatic drugs were not successful [20]. In a rituximab-allergic child ofatumumab, a fully humanized anti-CD20 antibody, has been examined by Pranzatelli et al. and was associated with favorable outcomes and eliminated CSF B-cell expansion [21]. OMS in our case was refractory to conventional treatments, IVIG, and also standard chemotherapy, but positive responses were observed with the first dose of rituximab.

Severity of disease based on the OMS Evaluation Scale dramatically decreased 6 months after treatment. In consistence with our result, Pranzatelli et al. also reported a case of OMS who showed marked clinical responses to rituximab [8]. In another study by this group rituximab was used as an adjunctive therapy to ACTH and/or IVIG. After rituximab they found that in 81% of cases lower motor severity score decreased and mean total score decreased by 44% [9].

Besides clinical responses, rituximab is associated with immunological responses in OMS. Relative B cell expansion in CSF has been proposed as a reliable biomarker for OMS [22]. Mean percentage of CD19+ B cells in CSF, but not in the serum, is higher in patients with OMS compared with controls [22]. Rituximab can afford long-term protection against CSF B cell expansion in OMS [10]; however, the main mechanism is not clear yet. Clinical effects of rituximab may be attributed to the removal of B cell as antigen-presenting cells than acting humorally mediated [23].

CSF/serum B-cell activation factor (BAFF) has been found to be higher in patients with untreated OMS compared with those in noninflammatory neurologic patients [24]. Pranzatelli et. al found a dramatic change in serum BAFF even after the first infusion of rituximab which revealed the probable role of BAFF in OMS activity [25]. Despite the wide investigations, the exact underlying mechanism of OMS is still unclear; moreover, the immunological pathway of rituximab is not completely diagnosed and all proposed mechanisms are just based on case reports. Prospective studies with larger sample size and adequate follow-up periods are highly recommended. It is also important to consider that the best treatment option is still under question. Since we did not perform immunological studies in our case before starting treatment with rituximab, we were not able to compare immunological changes.

## 4. Conclusion

Our case confirmed the clinical efficacy and safety of rituximab in a refractory case of OMS to surgical resection and conventional treatment.

## Figures and Tables

**Table 1:** Effects of rituximab on motor performance: a comparison of OMS evaluation scale scores before and after treatment.

Scale items	Before treatment*	After treatment*
(1) Walking, side-to-side imbalance	3	1
(2) Walking, front-to-back imbalance	3	0
(3) Walking, wide base	3	1
(4) Instability while standing (feet apart)	3	0
(5) Difficulty achieving standing position	2	0
(6) Truncal instability while sitting	2	0
(7) Targeting difficulty	3	1
(8) Difficulty grasping with one hand	1	1
(9) Difficulty with pincer grasp	2	0
(10) Abnormal eye movements while tracking (fixation)	2	0
(11) Abnormal eye movements while resting	2	0
(12) Speech abnormality (dysarthria)	2	2

*Scores were from 0 to 3 (0: normal, 1: mild, 2: moderate, 3: severe).
